# Using formative research to inform a mental health intervention for adolescents living in Indian slums: the ARTEMIS study

**DOI:** 10.1186/s13034-024-00704-4

**Published:** 2024-01-20

**Authors:** Ankita Mukherjee, Sandhya Kanaka Yatirajula, Sudha Kallakuri, Srilatha Paslawar, Heidi Lempp, Usha Raman, Beverley M. Essue, Rajesh Sagar, Renu Singh, David Peiris, Robyn Norton, Graham Thornicroft, Pallab K. Maulik

**Affiliations:** 1 The George Institute for Global Health, New Delhi, India; 2 Centre for Rheumatic Diseases, Faculty of Life Sciences & Medicine, King’s College London, London, UK; 3 Department of Communication, University of Hyderabad, Hyderabad, India; 4 Institute for Health Policy, Management and Evaluation, University of Toronto, Toronto, Canada; 5 Department of Psychiatry, All India Institute of Medical Sciences, New Delhi, New Delhi, India; 6 Young Lives India, New Delhi, India; 7 The George Institute for Global Health, UNSW Sydney, Sydney, Australia; 8 The George Institute for Global Health, Imperial College London, London, UK; 9 Centre for Global Mental Health and Centre for Implementation Science, Institute of Psychiatry, Psychology and Neuroscience, King’s College London, London, UK; 10 University of New South Wales, Sydney , Australia

**Keywords:** Child and adolescent mental health (CAMH), Depression, Formative research, LMIC, Self-harm, Slums, Stigma, mHealth

## Abstract

**Background:**

Adolescents are vulnerable to stressors because of the rapid physical and mental changes that they go through during this life period. Young people residing in slum communities experience additional stressors due to living conditions, financial stress, and limited access to healthcare and social support services. The Adolescents’ Resilience and Treatment nEeds for Mental Health in Indian Slums (ARTEMIS) study, is testing an intervention intended to improve mental health outcomes for adolescents living in urban slums in India combining an anti-stigma campaign with a digital health intervention to identify and manage depression, self-harm/suicide risk or other significant emotional complaints.

**Methods:**

In the formative phase, we developed tools and processes for the ARTEMIS intervention. The two intervention components (anti-stigma and digital health) were implemented in purposively selected slums from the two study sites of New Delhi and Vijayawada. A mixed methods formative evaluation was undertaken to improve the understanding of site-specific context, assess feasibility and acceptability of the two components and identify required improvements to be made in the intervention. In-depth interviews and focus groups with key stakeholders (adolescents, parents, community health workers, doctors, and peer leaders), along with quantitative data from the digital health platform, were analysed.

**Results:**

The anti-stigma campaign methods and materials were found to be acceptable and received overall positive feedback from adolescents. A total of 2752 adolescents were screened using the PHQ9 embedded into a digital application, 133 (4.8%) of whom were identified as at high-risk of depression and/or suicide. 57% (*n* = 75) of those at high risk were diagnosed and treated by primary health care (PHC) doctors, who were guided by an electronic decision support tool based on WHO’s mhGAP algorithm, built into the digital health application.

**Conclusion:**

The formative evaluation of the intervention strategy led to enhanced understanding of the context, acceptability, and feasibility of the intervention. Feedback from stakeholders helped to identify key areas for improvement in the intervention; strategies to improve implementation included engaging with parents, organising health camps in the sites and formation of peer groups.

**Trial Registration:**

The trial has been registered in the Clinical Trial Registry India, which is included in the WHO list of Registries, Reference number: CTRI/2022/02/040307. Registered 18 February 2022.

**Supplementary Information:**

The online version contains supplementary material available at 10.1186/s13034-024-00704-4.

## Background

Globally prevalence rates for diagnosed mental disorders among adolescents (10–19 years) is estimated to be about 13% [1]. Almost half of all adult mental disorders manifest before 14 years of age and about 75% by 24 years [2]. India has the largest adolescent population in the world, about 253 million [3, 4]. A national survey found the prevalence of mental health morbidity among adolescents (aged 13–17 years) to be 7.3% [5]. Self-harm is a leading cause of death contributing to about 10% of all deaths among boys and 16.8% deaths among girls aged 15–19 years [6].

The Adolescents’ Resilience and Treatment nEeds for Mental health in Indian Slums (ARTEMIS) study, a cluster randomized control trial (cRCT), will implement an intervention intended to improve mental health outcomes of adolescents living in slums in India [7].

## The ARTEMIS cluster randomised controlled trial

The ARTEMIS study aims to develop and evaluate an intervention that delivers mental health care to adolescents with depression, increased risk of self-harm/suicide, or other significant emotional or medically unexplained complaints among adolescents living in urban slums in two cities in India. In ARTEMIS, the definition of suicide and self-harm adhere to the definition given in WHO’s mhGAP tool [8], wherein, suicide is the act of deliberately killing oneself. Self-harm is a broader term referring to intentional self-inflicted poisoning or injury, which may or may not have a fatal intent or outcome. The study sites are New Delhi, located in north India, and Vijayawada located in south India. The study is being conducted in 60 slum clusters spread across the two cities and has two core components [7]. The first is an anti-stigma campaign that aims to improve community behaviours towards adolescents with depression or at increased risk of self-harm/suicide. The second is a technology-enabled mHealth intervention using an electronic decision support system (EDSS) to help primary health workers, including non-physician health workers (known as Accredited Social Health Activist or ASHAs) and urban primary health centre (UPHC) doctors to diagnose and treat adolescents who are at high-risk of depression, increased risk of self-harm/suicide.

In this paper we report the results of the formative research phase that has informed the development of the intervention for the main ARTEMIS trial.

## Rationale

A mixed methods formative evaluation was undertaken to improve the understanding of site-specific context, assess feasibility and acceptability of the two components and identify required improvements to the intervention. In-depth interviews and focus groups with key stakeholders (adolescents, parents, community health workers, doctors, and peer leaders), along with quantitative data from the digital health platform were analysed.

### Aims and objectives

The formative research was undertaken with the following aims:

1. To obtain information on the local context (including key stakeholders), factors influencing the uptake of mental health services and common stressors among adolescents living in the study area.
2. To assess the feasibility of rolling out a community-based anti-stigma campaign and the acceptability of anti-stigma IEC content among adolescents, and
3. To assess the feasibility of rolling out the mHealth component of the intervention and acceptability of the EDSS among ASHAs and UPHC doctors.

## Methods

The UK Medical Research Council (MRC) guidance for developing and evaluating complex interventions [9] has informed the formative phase of ARTEMIS. This formative study was conducted in a large slum in New Delhi (a block of Prem Nagar III) and three smaller slums in Vijayawada (Kreesthurajapuram, Karmika Nagar and Gangiredduladibba) with a population of approximately 1400 in each city. This population target size was selected to identify at least 50 ‘high-risk’ adolescents per site. The slums selected for the formative study will not be included in the subsequent implementation of the main trial. Each site had one UPHC led by a doctor.

**Fig. 1:**
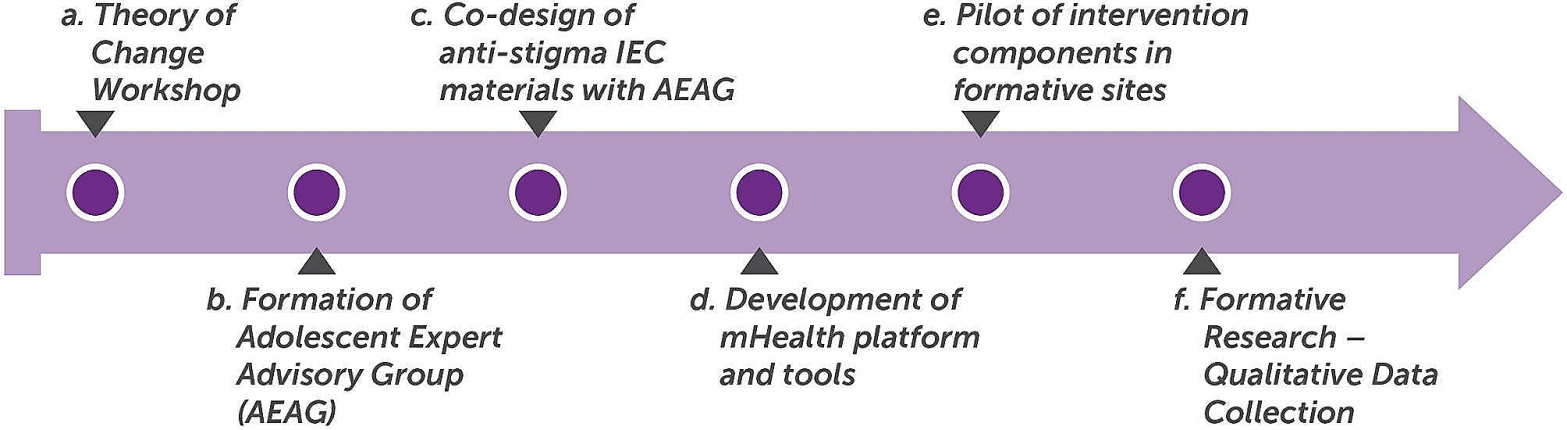
Research Activities in the Formative Phase of ARTEMIS

The ARTEMIS team undertook several stages of activities in the formative phase (Fig. 1).

Research activities included meetings, consultations, and workshops with key stakeholders - adolescents, parents, doctors, community health workers, and subject matter experts - to develop a theory of change (ToC) for the intervention. It also included co-designing the anti-stigma information education communication (IEC) materials with adolescents and developing the mHealth platform and tools.

After the intervention roll out, in which the anti-stigma campaign and technology enabled EDSS were piloted, a formative evaluation was undertaken using both quantitative and qualitative data methods.

The quantitative data from the EDSS used for screening and diagnosis was collected directly from the server located at our office in Hyderabad. The qualitative study included 11 focus group discussions (FGDs) with 99 participants and 19 in-depth interviews (IDIs) across both sites (Table 1). Participants were selected purposively [10]. A baseline survey tool was administered to 50 adolescents to test the comprehension and relevance of questions. Feedback on the baseline tool was collected during the qualitative study.

**Table 1:** Data sources for qualitative study

Participants	FGDs	Interviews
Adolescents (including ‘High-Risk)	7	6
Parents of ‘High-Risk’ Adolescents	-	11
PHC Doctors	-	2
ASHAs	2	-
Peer Leaders	2	-
Total	11	19

The qualitative data were transcribed and translated into English. Framework analysis [11] involving inductive and deductive techniques was used, wherein themes were developed both inductively from the responses of research participants as well as from information related to specific pre-decided thematic areas. The researchers wanted to understand key issues related to acceptability, feasibility and possible changes needed for the future implementation of the two intervention components in the main trial. Discussions were held among the researchers to critically examine the responses of participants and arrive at an agreement on the recurring themes. The data was coded using NVivo and the following codes emerged:

1. Adolescent Stressors
  1.1  Career related stress
  1.2  COVID induced stress
  1.3  Domestic quarrels
  1.4 Financial problems
  1.5  Forced marriage & early marriage
  1.6  Gender related stressors
  1.7  Love affairs
  1.8  Parental attitudes
  1.9  Sanitation, water-logging
  1.10  Substance use-abuse
2. Anti Stigma Campaign
  2.1  Games feedback
  2.2  Lived experience videos feedback
  2.3  Magic show feedback
  2.4  Print material-posters feedback
  2.5  Seminar feedback
  2.6  Street Play feedback
  2.7  Suggestions about anti-stigma activity
  2.8  Takeaway messages from anti-stigma campaign
3. ASHA follow-up- Experiences, Challenges
  3.1  ASHA follow up- high workload
  3.2  ASHA follow-up- difficulty in talking about suicide
  3.3  ASHA follow-up- parental worry, fear stigma
  3.4  ASHA follow-up-explaining to adolescents about their mental health
  3.5  ASHA followup-dealing with neighbours
4. Context
  4.1  Crime, illegal activities
  4.2  Education, employment, socio-economic context
  4.3  Local politics, policies, local administration
  4.4  Location-physical surrounding
5. Coping with stress
6. Doctor treatment experience
7. EDSS-Tab usage
  7.1  EDSS-Tab usage – challenges
  7.2  EDSS-Tab usage- feedback and suggestions
8. Overall feedback on programme
9. Potential barriers
10.  Potential facilitators
11.  Reason for parental co-operation
12.  Reasons for not seeking care.
  12.1 Already under treatment from elsewhere
  12.2  Distance from UPHC, Transport facility
  12.3  Fear of being institutionalised
  12.4  Parental Refusal, Stigma
  12.5  School timings
  12.6  Self Stigma
13. Training
14. Understanding mental health

Once all the data had been coded it was collated, and analysed using thematic content analysis using NVivo 12. Quotations that were interesting and illustrative of a theme were highlighted and used in the analysis.

## Results

The results are presented in two sections. Firstly, the research activities which led to development and refinement of the intervention are detailed, and secondly results from formative testing of the intervention components are presented.

### Development of tools and intervention materials

#### Theory of Change (ToC) workshop

**Fig. 2:**
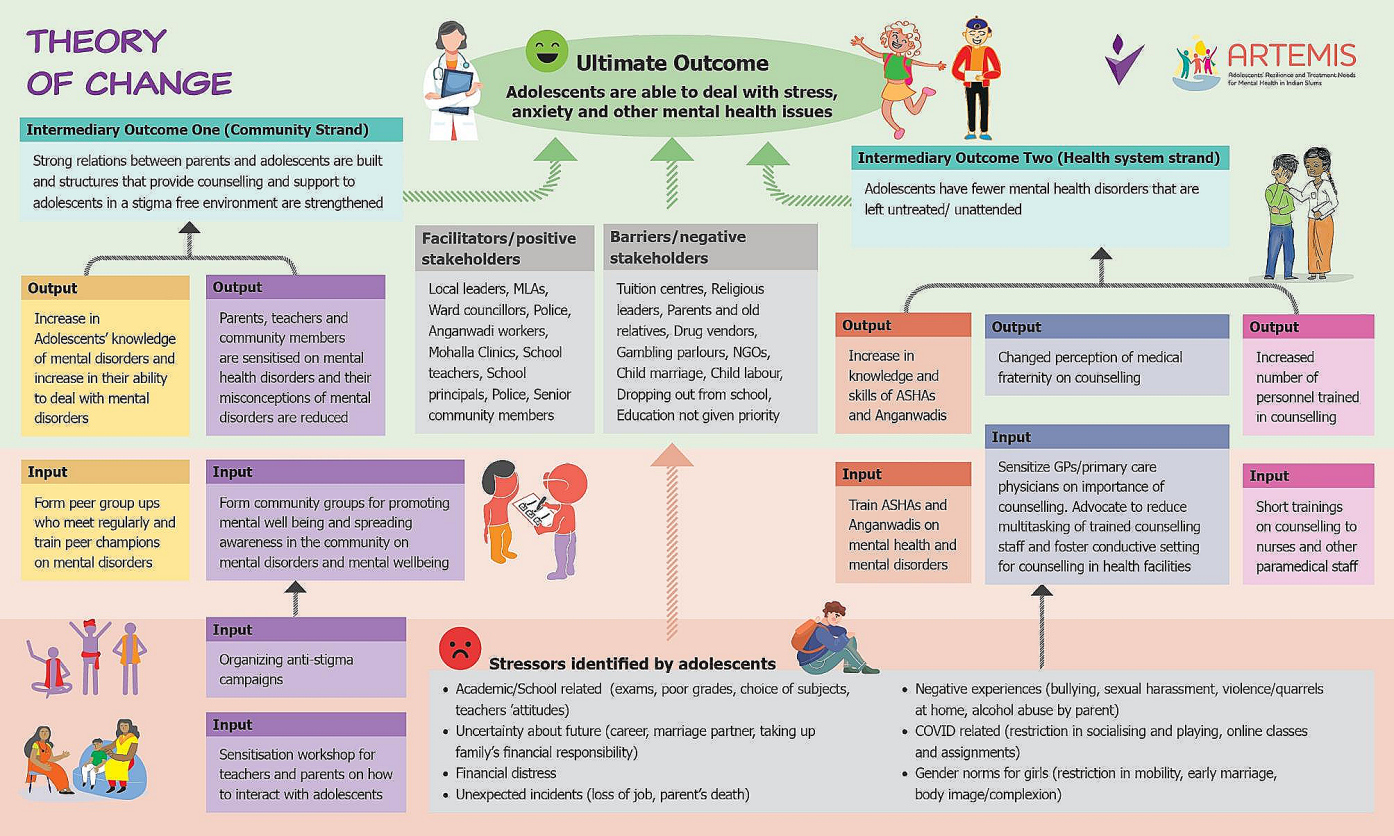
ARTEMIS Theory of Change (ToC)

As part of the formative phase, a ToC [12] workshop was organised in three phases to help develop a programme theory for the intervention (Fig. 2). Participants were purposively selected in each phase to include a range of stakeholders engaged at the community and policy levels. Phase 1 included interaction with adolescents aged 15 to 19 years, parents of adolescents aged 10 to 19 years, ASHAs and *Anganwadi* workers (women who manage the government-run early childhood care centres in the locality). Phase 2 engaged doctors from UPHCs and local elected representatives. Phase 3 consisted of discussions with academics with expertise in policy making. The ToC aimed to explore pathways that the project could adopt to address stressors faced by adolescents in their everyday life. This approach helped to identify local stakeholders, potential facilitators, and barriers for implementation of the intervention and the policy context related to child and adolescent mental health across the two cities [13]. Suggestions were sought from stakeholders on possible strategies to increase the uptake of mental health services by adolescents.

#### Formation of adolescent expert advisory groups

The **Adolescent Expert Advisory Groups** (AEAGs) are a vital component to the project and its role was to inform the researchers about anti-stigma components and the best way to implement them. A group of adolescents from each study sites constituted the AEAGs. Initially, 3–4 engagement meetings were conducted (October-December 2020) with adolescents from each study site, to build rapport and identify adolescents who would serve on the AEAGs. The adolescents were selected based on their response during the engagement meetings – attendance in all 4 meetings, active participation in the discussions, and taking part in the activities assigned to the group [14]. Based on these interactions, three AEAG groups were formed in each site. This included a mixed group of boys and girls in the age group of 10–14 years (referred to as ‘younger adolescents’). Separate groups for boys and girls in the age group of 15–19 years (referred to as ‘older adolescents’) were also formed. Parental consent and assent for underage participants and consent for adolescents over 18 years was taken before formalising their membership [14].

#### Co-designing anti-stigma IEC material with adolescent expert advisory groups

For the intervention, the aim was to design an anti-stigma campaign that was acceptable to adolescents. This was achieved through co-designing the materials to ensure that they were adolescent-friendly, contextually relevant, drew on and reflected the experiences of adolescents living in slum communities.

A total of 34 meetings were organized over a 10-month period (January 2021 to October 2021) with AEAG members (19 meetings with older adolescents and 17 meetings with younger adolescents). These meetings were facilitated by researchers (SKY, SK, SP) from the project with support from other project staff. In the first few meetings the aim was to acquaint the AEAG with the project, identify common stressors faced by adolescents that impact their mental well-being, understand their current coping strategies, and discuss experiences of stigma related to mental health. In subsequent meetings, strategies to deliver a community-based anti-stigma campaign and the content for campaign materials were discussed. The key suggestions made by AEAG members were discussed by the researchers and were subsequently used to develop resource materials and adopt implementation strategies. These were then discussed with the AEAGs iteratively and refined. AEAG feedback was also sought to identify ways in which to engage with other adolescents in the area and their parents.

The following IEC materials were developed in the Hindi and Telugu languages.

##### *C(i)* interactive games with mental health messages

Games were viewed by the AEAG as the most popular way of reaching out to adolescents. In Delhi site ‘snakes and ladders’ was suggested and in the Vijayawada site adolescents suggested using the popular sport of cricket as a theme to develop a board game. Younger adolescents and girls proposed ‘hopscotch’ as a popular game. These suggestions were taken up by the research team. Mental health related messages were integrated into the design of these games. For example, in snakes and ladders all behaviours promoting mental wellbeing (such as sharing problems faced or sharing experiences) were printed at the base of ladders, while behaviours that had an adverse impact on mental wellbeing (such as consuming intoxicants in large amounts) were printed at the head of the snakes. The games were played by AEAG members, and the messages were finalised based on their feedback.

##### *C(ii)* videos of adolescents with lived experience

Direct exposure to stories shared by persons with lived experience (PWLE) or their caregivers (social contact) is the most effective anti-stigma intervention [15, 16], Adolescents from the community, and the AEAG members volunteered to record their experience for four short videos for the campaign. Topics included substance use, stress due to early marriage, stress due to gender stereotyping and the stigma faced by family members of people living with mental illnesses.

##### *C(iii)* posters

Posters with messaging that promoted help-seeking, highlighted symptoms of depression and coping strategies, encouraged adolescents to reach out to one’s social support networks such as parents, teachers, and friends were designed in consultation with the AEAG.

##### *C(iv)* street play

Street plays were developed with AEAG inputs and focused on stress and suicide ideation due to academic pressure, lack of parental trust, early marriage, and the role of parents in facilitating help-seeking. Adolescents from the community and AEAG members were trained to enact the street plays.

##### *C(v)* rally

AEAG members suggested holding rallies to spread awareness, trigger curiosity and interest on mental health in the slum community using catchy slogans and placards. The slogans were created by the AEAGs and refined by the researchers in consultation with them.

##### *C(vi) magic show*

A brainstorming session between AEAGs and the researchers resulted in the innovative suggestion of using magic shows to convey key mental health messages and promote help-seeking. Messages on supporting people with mental illness and challenging stigma around mental health were woven into the magic shows performed by a professional magician.

##### *C(vii) peer leaders*

Based on suggestions of the AEAG and discussions during the ToC workshops, four ‘peer leaders’ between 17–19 years were identified by research staff in each site (two males and two females). Their role was to provide support to adolescents who approached them and to promote mental health awareness in the community. They were provided with basic training on mental health, addiction and substance use, digital health, staying safe on social media, dealing with COVID–19 and promoting mental wellbeing.

#### Developing technology enabled EDSS platform and tools

A technology enabled EDSS platform was developed for screening, diagnosis and follow-up of adolescents screened as at high risk for depression and/or suicide. Patient Health Questionnaire-9 item (PHQ9) [17] integrated in electronic tablets was used as the screening tool for depression and suicide risk. Adolescents with an overall score of ≥ 10 and/or score ≥ 2 on the self-harm/suicide related question on the PHQ9, were considered as ‘screen-positive’ and at high risk for depression and/or suicide [17]. This group will be referred to as ‘high-risk’ in this paper. The EDSS developed for UPHC doctors, integrated WHO’s mhGAP-IG [8], providing an algorithm to enable diagnosis, treatment or referral of adolescents needing mental health care. The EDSS was based on a similar platform for adults developed for the SMART Mental Health Project in India [18, 19]. The platform was adapted for adolescents; behavioural and developmental disorders that were appropriate for adolescents were included. Once an adolescent visited the doctor, the EDSS alerted the ASHAs about the status on a real-time basis on the dashboard, which had a traffic light system to help ASHAs to prioritize adolescents who needed follow-up.

#### Piloting implementation (Sep-Oct 2021)

The anti-stigma campaign, and the technology enabled EDSS were pilot tested together in the formative sites. A baseline tool was also piloted with adolescents and their parents, to check for understandability, and relevance to adolescents. The baseline tool captured sociodemographic characteristics, social support, stressors, resilience, attitudes, and behavior related to mental health, stigma related barriers to access care, history of mental disorders and service use, and economic evaluation for those who had used mental health services. Field investigators (responsible for collecting data) were trained to use the baseline tool and data was collected using hand-held tablets.

##### *E(i) Piloting the Anti-Stigma Campaign*

To reach out to high-risk adolescents in the limited time available, a one-day event called the ‘*Adolescents’ Fair’*, was organized in both sites. About 100 adolescents in both Delhi and Vijayawada participated in it. During the fair, feedback was taken from the participants on the various anti-stigma elements, using voting charts with smiley faces. Besides, community members and adolescents were exposed to anti-stigma campaign activities in the formative sites (Table 2).

**Table 2:** Frequency of anti-stigma campaign activities

Campaign activity	Total number organised (includes both sites)
Adolescent Fair	2
Street Play	25
Rally	3
Games	38
Community Meetings	82

##### *E(ii) Technology-enabled EDSS*

Handheld tablet devices were used for screening, diagnosis, and management of conditions. Trained field staff used the PHQ9 to screen all adolescents in the formative sites to identify high-risk adolescents. The UPHC doctors in both sites were provided with half day training on diagnosis and treatment of depression and suicide risk in adolescents and in the use of the EDSS. The training was done by the principal investigator, a trained psychiatrist.

The ASHAs in each site received training for three days by the researchers on adolescent mental wellbeing, and use of the EDSS to follow-up high-risk adolescents. The ASHAs were supported for 10 days post-training by trained field staff to deal with any problems that they faced while navigating the application initially. Competency testing was done for both ASHAs and doctors following the training.

The ASHAs followed up the identified high-risk cohort, to encourage them to consult the UPHC doctor. The doctor examined the high-risk adolescents who visited the UPHC using the EDSS tool. Adolescents who required specialist intervention were referred to a psychiatrist at the nearest public hospital.

Additionally, two health camps (out-patient clinics organized in the community as opposed to in the UPHC) were organized in Vijayawada where the doctor visited the slums, and the adolescents were able to consult them closer to home.

Since we were working with PHC doctors and the ASHAs, we provided them with a fixed incentive which is in line with the government system. The Adolescent Expert Advisory Group members were reimbursed travel expenses, as were participants of the theory of change workshop. However, no other incentives were provided to the research participants or their families.

### Formative evaluation of the intervention

A total of 2752 adolescents were screened in the formative sites across both cities, of whom 133 (4.8%) were identified to be at high risk. The findings are discussed below in relation to the objectives of this formative research study.

#### The intervention context

##### Living conditions, physical context and availability of mental health services

In Delhi, the slum was built on unauthorised low-lying agricultural plots with no planned sewage system. There was continuous water logging and poor sanitary conditions. This was identified as one of the factors affecting the quality of life and well-being of adolescents.

*There is no sewerage system over there as of now. …. 20–40% of the streets over there are such where it is totally impossible for you to go in. They [slums] are filled with sewerage 24 × 7 – Staff FGD, Delhi*.

*After returning from office, if there is a drain full of water in my street, then obviously there are some activities that I wouldn’t be able to do, such as meeting my friends, roaming around in the evening, going out to the markets. –Staff FGD, Delhi*.

In some areas the local administration had elevated the street levels with the result that the ground floor in many houses had doors which were below street level which caused rainwater to drain into those floors and damage property.

On the other hand, the slums identified for the formative phase of the project in Vijayawada were notified slums with a proper sewerage system. However, they were located on hills that made the area difficult to traverse and many houses could be accessed only through rough steep steps.

Based on a facility mapping and discussion with local staff, it was found that the availability of public mental health services was limited in the study areas. In Vijayawada, mental health services were available in the government general hospital, which was about 10 km away from the slum cluster selected for the formative research. A government programme for adolescent friendly public clinics to provide weekly counselling services is not yet operational in Vijayawada. In Delhi there were several public hospitals but the tertiary hospital closest to the site did not have mental health services. While some of the adolescent friendly clinics were operational, adolescents in the area were not aware of these services. Private practitioners were available in both sites, but the costs were out of reach for families living in the slums.

##### Common stressors among adolescents

Adolescents spoke of several stressors in their day-to-day life. These included studies and grades, worries about future responsibilities like supporting the family financially, financial distress and quarrels among parents.

*Participant 1: If we don’t pay the [school] fees they remove (expel) us from school…….*

*Participant 2: At such times, we feel like we should stop studying. ….*

*Participant 3: I would also feel the same way. Because we will not have money to buy books. If we stop studying at least that money will be used for some other purpose. – Adolescent FGD 3, Vijayawada*.

*If we don’t pay rent, they will tell us to vacate the house. … they will throw all our things [furniture, utensils, clothes etc.] outside- Adolescent FGD 3, Vijayawada*.

*Participant 1: I feel stressed when a fight takes place in the house.*

*Participant 2: If I get less marks in exams, then also I feel tensed.*

*Participant 3*: *I worry what would happen if I failed [exams]. -Adolescent FGD 1, New Delhi*.

Girls in both sites spoke about their worry of being pressured to get married before they were ready or had completed their education.

*Participant: Stress about marriage… Means when parents get us married at a young age. At a young age they make us quit our studies and get us married. - Adolescent FGD1, Delhi*.

##### Parental role and involvement with adolescents

Many of the adolescents were children of migrant blue-collar workers, informal home-based workers, or daily wage labourers. The main bread-earner, mostly the father, often had to work long hours to make ends meet and was hardly home. Occasionally both parents worked long-hours.

*He (Father) returns home around 10:00 p.m. He comes back late, and then he leaves early in the morning around 08:00 a.m.- Parent Interview 2, Delhi.*

This situation also meant that many of the parents were unable to take part in the community-based anti-stigma activities or discuss the activities that their children had engaged in during the adolescent fair.

*A play was also conducted over here…. I did not pay so much attention to that, but they were telling something related to… (tries to remember) the word health was written there… Parent Interview 2, Delhi*.

*No ma’am I didn’t attend (community meetings). Madam (project staff) came it seems, but I was not there at the time. I had gone out somewhere. -Parent Interview 6-Vijayawada.*

Several adolescents reported challenges in discussing difficult or stress-inducing topics with their parents, such as scoring poorly in exams or sexual harassment. This could be due to fear of being reprimanded or withdrawing them from school/college.

*If anyone [boys] harasses [girls] in college, and if they share it [harassment] with their parents, they [parents] will ask her to stop going to the college… We should not share such things [incidences] with our parents*. Adolescent FGD 1, VJA.

Adolescents were extremely resistant to discussing any self-harm ideas with parents [20]. They did not view parents as someone they could confide in. Parents themselves were sometimes the main source of stress among adolescents, and due to the lack of frank discussion between them, some adolescents had contemplated or attempted suicide.

*The stress I found here for older patients [adolescents] mostly were related to parents scolding or fighting…I remember 4–5 adolescents had planned or thought of suicide. When asked why, then [they answered] because mom scolded that is why I thought of it[suicide]. – Doctor interview -Delhi*.

#### Feasibility and acceptability of anti-stigma campaign components

There was overall positive feedback about the anti-stigma campaign. Adolescents recalled the activities they participated in, and the audio-visual content shown to them. Younger adolescents recalled most of the activities and narrated the stories in the street play and videos. Older adolescents were able to articulate their psychological symptoms as well as the need to share their feelings with trusted adults following the campaign. When asked about key learnings and take-home messages from the campaign, they were able to articulate these well.

*We should not take any mental stress. In case we feel that [stress] we should discuss about that at home with someone or take help from friends. If our friends are not able to help and other people around us are also unable to help… you should take help from people around, you. Adolescent FGD2, Delhi.*

*Younger kids will be attracted more to games. So that is the right way to convey messages. While playing the game the kid should read the message and explain it, that will be helpful [in conveying the message] and it creates interest. – Adolescent FGD 7, VJA*.

The peer leader initiative was not viewed positively in Delhi. Adolescents reported that they did not approach them and rarely remembered their names. In discussion with peer leaders, they confirmed the same. They gave feedback that there was a need to spread more awareness about their role and responsibilities in the community.

*“Not even a single adolescent has visited till now to interact*.” – *FGD, Peer Leaders, Delhi*.

*“Maybe they [adolescents] think that we [peer leaders] will not be able to provide any relevant solutions”* -*FGD, Peer Leader, Delhi*.

In Vijayawada two adolescents approached the peer leaders. The first was a girl whose parents wanted her to get married against her will. The peer leader listened to her and told her that, if needed, the project staff would talk to her parents. The second was the neighbour of a peer leader who was upset because her parents would often scold or beat her. The peer leader talked to the adolescent and her mother.

#### Feasibility of implementing technology-enabled mHealth component

##### Use of EDSS for screening, follow-up, and treatment

The EDSS was used to screen 2752 adolescents (1321 in New Delhi, 1431 in Vijayawada). Table 3 provides data from both sites combined. Gender amongst those screened was almost equal. Across both sites, 133 adolescents were at high-risk; the screen positive rate in Delhi and Vijayawada was 5.98% (*N* = 79) and 3.77% (*N* = 54) respectively. The adolescents who were screened positive were followed up by ASHAs, who advised parents to seek care for their children from the UPHC. In both sites, all high-risk adolescents except one (who was unavailable despite repeated visits) were followed up at least once by the ASHAs.

**Table 3:** Profile of participants (*N* = 2752)

Gender	Male	1367 (49.7%)
	Female	1384 (50.3%)
Age	10–14 yrs.	1354 (49.2%)
	15–19 yrs.	1362(49.5%)
Screened status	High Risk	133 (4.8%)
	Non-High Risk	2619(95.2%)

**Table 4:** Treatment and Follow-up of ‘high risk’ adolescents (*N* = 133)

Number of male high-risk adolescents	64 (48.8%)
Number of female high-risk adolescents	69 (51.2%)
High risk adolescents in age group 10–14 years	54 (40.6%)
High risk adolescents in age group 15–19 years	79 (59.4%)
High risk adolescents with depression score ≥ 10	95 (71.4%)
High risk adolescents with suicide score ≥ 2	67 (50.3%)
Number of high-risk adolescents followed up at least once by ASHAs	132 (99.2%)
Number of high-risk adolescents who were seen by the UPHC doctor	75 (56.39%)
Number of high-risk adolescents who were referred to a psychiatrist	9 (6.7%)

In all 56.4% (49.4% in New Delhi and 66.7% in Vijayawada) of the ‘high-risk’ adolescents visited the PHCs and sought help for their mental health symptoms over the 2-month formative study period. (Table 4).

Both doctors and ASHAs were able to navigate the EDSS easily and did not report any challenges in using the tablet device.

*I had never used it (tab) before, but I had no problem using it according to the instructions I was given. -* ASHA FGD Delhi.

However, the doctors and ASHAs felt uncomfortable talking about suicide and self-harm risk.

*In my opinion, it is not necessary to ask the suicide question to every adolescent…. So many kids used to get shocked. Used to say - what are you asking us? Suicide? Why would I [want to] commit suicide? Why would I do such a thing?* - Doctor, Delhi.

*There’s a question which asks, “do you feel that you want to die sometimes, or you want to live?” They [parents] used to feel very bad about this question.* ASHA FGD-Delhi.

Similar feedback was provided by some ‘high-risk’ adolescents who mentioned that they felt uncomfortable when they were asked questions related to suicide. One adolescent who had visited the UPHC mentioned:

*The dispensary that we had visited, the doctor there was asking weird questions. He was talking about things that people would not feel like doing. … I found all this very wrong… He asked, what do you feel like doing, like hanging yourself or committing suicide, who troubles you the most at home. I found those things to be bad.* High Risk Adolescent (female) Interview-1, Delhi.

This highlighted the need for focusing on appropriate ways to ask about suicide in our trainings with doctors.

##### Challenges in help seeking

Stigma was found to be a major barrier to help-seeking. Parents refused to accept that there was ‘anything wrong’ with their child and many parents refused to send ‘high-risk’ children to the UPHC. There were also misconceptions among parents about the kind of mental health treatment their children would receive.

*They [parents] want to know about treatment, how it will be, will they give shock treatment*… – *ASHA FGD, Vijayawada*.

*They [parents] told me - …. we are concerned what others will think about our kids, when we bring them [to the doctor] … so we don’t send the kids. - ASHA FGD, Vijayawada*.

*The parent said, “what is the use of giving medicine to my child, what is the use? You simply bother our children, what is the advantage of it [sending child to doctor]? What benefit do we get because of this [treatment].” She [daughter] was referred to A________ (tertiary hospital), but she [parent] refused*. - *ASHA FGD, Delhi*.

There were practical challenges in accessing care also. Since UPHC opening times often overlapped with school schedules, adolescents had difficulty in visiting the UPHC, especially in Vijayawada, where the UPHC was located at a greater distance from the slums. This problem was addressed by organising two camps in Vijayawada. Out of 26 adolescents who were seen by the doctor, 14 came to the health camps organised in the slum. In Delhi no health camps were held in the community, as the UPHC was nested in the slum itself.

## Discussion

### Summary of findings

The UK Medical Research Council (MRC) guidance for developing and evaluating complex intervention [9] has informed the formative phase of ARTEMIS. The MRC guidance highlights the need to assess feasibility and acceptability of an intervention after identifying the intervention. Core elements in understanding and assessing feasibility need to include consideration of context, developing and refining programme theory, engaging with stakeholders, identifying key uncertainties, refining the intervention and economic considerations. For instance, using peer leaders as a point of contact to provide lay counselling and mental health support for adolescents in the community was not part of the original plan. However, since this came up strongly in the theory of change workshops, it was introduced into the intervention. However, the formative phase showed us that peer leaders were not acceptable to the adolescents. After intense deliberation between the researchers, implementation team and the AEAG, it was decided that instead of having two peer leaders per intervention cluster, we would form a peer group comprising of 10 to 15 adolescents instead. Thus, the formative phase helped to locally tailor and design key components of the intervention. The modifications made to the intervention based on findings of the formative research have been briefly summarised in the Table 5.

**Table 5:** Summary of adaptations made/proposed to ARTEMIS based on formative research findings

Findings	Adaptations/inclusions to the intervention
**Anti-Stigma Campaign**	
Adolescents showed interest in audio-visual and game-based methods for the anti-stigma campaign.	Interactive games which integrate anti-stigma messages were co-designed with the help of adolescents. The research team discarded some ideas like use of comic strips as they were not popular with adolescents. Audio dramas have been added to the anti-stigma IEC material based on suggestion from AEAG.
Academic pressure, parental expectations, fights between parents, substance use, and financial problems were important stressors. Gender based norms like restriction in mobility, inability to continue education or early/forced marriage were important stressors among older adolescent girls.	Street plays and audio drama stories based on themes that highlight some of the stressors faced by the adolescents have been produced. The street plays and audio dramas will provide an opportunity to discuss sensitive issues such as early/force marriages and possible negative consequences of such practices on adolescent mental and physical wellbeing. These will set the stage to enable more detailed discussions with the adolescents themselves, their parents and members of the community.
Parents were identified as important stakeholders who need to be engaged during the intervention. There was difficulty in communication between children and parents on mental health issues. Parental stigma was an important barrier for not seeking care.	A key activity at the start of the intervention will be to meet and actively engage with parents through meetings. Parents of ‘high-risk’ adolescents to be specifically targeted in the anti-stigma campaign that will also be held on holidays and Sundays to increase participation. Some of the anti-stigma campaign activities will target parents. For instance, the important role that parents could play in recognising symptoms of distress and depression in adolescents and facilitating help seeking, will be demonstrated through a street play. The street play will be followed by a discussion where the need to normalise mental disorders, overcome stigma associated with mental disorders and actively seek help will be reinforced by the implementing team.
Younger adolescents in the age group of 10–12 year recalled activities but were sometimes not clear on the takeaway message.	A list of key takeaway messages for each activity will be prepared. Field staff will be trained to deliver these messages after each activity.
***mHealth***	
Questions related to suicide and pregnancy in the baseline survey tool and EDSS tool caused discomfort among adolescents. The baseline tool took too much time (an hour) to administer.	Question related to pregnancy was retained only for married adolescents. Training of field staff, community health workers and doctors to focus on sensitive ways to approach suicide related questions. For instance, during the training using the WHO mhGAP tool [8], doctors will be reassured that enquiring about suicide ideation will not trigger suicide in adolescents susceptible to or at risk of suicide; rather such an enquiry might help to reduce the ‘anxiety associated with thoughts or acts of self-harm and help the person feel understood’ [8] and prepare them to navigate such situations. Regular monitoring and check-in with doctors by the research team are planned to ensure doctors ask these questions appropriately. The baseline tool was shortened.
Adversities related to living in the slum context was an important stressor which the baseline tool did not capture	An additional section was added to the baseline questionnaire to capture impact of slum adversity on mental health of adolescents.
Several barriers to seeking help from doctors existed, including similar working hour of UPHC and school, distance from UPHC and inability of working parents to accompany their wards to the UPHC.	Community level health camps in which doctors would visit the slums, were planned for the intervention.
***Other***	
Stakeholders who could potentially support the intervention were identified.	In Vijayawada women’s self-help groups and community volunteers under a government programme were identified to help during the anti-stigma campaign.
The Peer Leader initiative was not found to be very popular. Adolescents were not familiar with the Peer Leaders and did not approach them.	The plan for engagement and role of Peer Leaders was revised. Instead of one or two Peer Leaders, peer groups will be formed. Peer group will be more involved in mobilising and interacting with adolescents during the anti-stigma campaign. Peer group members will be trained to increase their awareness about mental health and the importance of help-seeking. They will also be trained on how to communicate these to other adolescents in their slums using the anti-stigma materials developed by the research team.

### Comparisons with other studies

Findings related to local context helped us recognise the need to include local living conditions as a contributor to stress and mental health of adolescents living in vulnerable settings like slums. Studies in slums in other low-and middle-income countries (LMICs) have found slum related adversities to have a negative impact on the mental health of adolescents [21, 22]. Easy access to substances and exposure to violence in the home and neighbourhoods, domestic pressures to get married (especially for girls) and financial uncertainty were identified as key stressors. Other studies have also reported similar stressors amongst adolescents [23, 24].

Several mental health interventions have used ToC during intervention development, during the pilot phase and to refine the intervention [12, 25, 26]. In ARTEMIS too, the research team engaged with stakeholders to develop the ToC, which helped to identify goals, outputs and key programme inputs that would help achieve the outcomes. The ToC also helped to identify stakeholders who could potentially support the intervention. In Vijayawada, women’s self-help groups and community volunteers under a government programme were identified as potential stakeholders. It also led to the research team making adaptations. Suggestions regarding the need for individuals or groups who would be accessible in the community and with whom adolescents could communicate easily emerged during the discussions, which led to developing the peer leader/group initiative.

The engagement with the AEAG was critical in co-creating the anti-stigma campaign materials, which was a collaborative process that involved collective creativity and multiple iterations throughout the problem solving process [27–30]. Regular meetings with AEAGs helped to come up with acceptable anti-stigma campaign strategies for adolescents in the community to spread awareness about mental health. The content for the IEC material was guided by contributions and discussions with the AEAG. This process helped the research team to co-create an anti-stigma campaign that was contextually relevant and acceptable to adolescents. Earlier research has also underlined the importance of using co-designed interventions with adolescents [31–33] and they reported good acceptability amongst end users [34].

The mHealth component of the intervention was found to be feasible and acceptable among community health workers and doctors. While there has been a steady rise in studies testing digital health interventions for mental health in LMICs, there is limited availability of literature on effective mHealth interventions in the field of adolescent psychiatry in LMIC settings [35]. However studies on initial feasibility and acceptability of such interventions have shown promising results [36–39]. Prior research has demonstrated the EDSS to be useful in aiding clinical decision making in primary care settings and improving mental health outcomes [40–42]. Digital technology was found to be useful in low resource settings where the availability of mental health specialists is limited.

The formative research found that doctors need to be trained on appropriate and sensitive ways to approach suicide related questions. This is particularly relevant in the study context where adolescents reported hesitation to discuss suicide. Adolescents who were at risk of suicide did not want the researchers to share their suicide scores with parents. When discussing help seeking for their wards with parents, adolescents wanted us to refer to stress and not suicide or self-harm [20]. It is possible to link this with the stigma associated with suicide, which as pointed out earlier, is equated with doing something ‘wrong’ or ‘bad’. Parental attitudes reinforce this. Not only doctors and ASHAs, but field staff who screened the adolescents also perceived negative reaction from parents whenever questions related to suicide were asked. There was also the possibility that questions related to suicide were not asked sensitively to some adolescents. There is very little focus on psychiatry in undergraduate medical training [43] not only in India but in other high income countries also. This has implications for identifying patients at high risk including suicide risk [44], as well as the necessary skills required in asking questions related to sensitive topics like suicide.

Several barriers were identified which prevented high risk adolescents from seeking care. Mental health related stigma amongst parents, lack of time due to long work hours, and financial constraints of the families were important barriers. Almost all high-risk adolescents were followed-up by the community health worker, and their families were advised to visit the UPHC. However, only a little over half visited the UPHC doctor. While reluctance of parents due to stigma was a significant barrier, it is important to note that this formative project was a 2-month study and our prior experiences of working with adults showed that organizing health camps was the most effective way of increasing access to health care in the community. Most families lived far from PHCs, and did not want to spend money and time travelling there [39]. Other studies have highlighted the importance of stigma associated with mental health and poor knowledge about mental health and illness as barriers to care seeking [45–47]. Parents seem to conceal their child’s mental health condition, which contributes to self-stigma by adolescents themselves [48]. Further the findings showed that at times parents also appear to cause stress to their adolescent children. This highlights the need to include parents for any interventions targeting adolescent mental health. It is crucial to sensitize parents about mental health of adolescents and address stigma related to help seeking.

## Conclusion

These research activities during the formative phase of ARTEMIS increased the research team’s understanding of the intervention context and provided useful insights which informed and improved implementation methods and strategies. The formative phase helped to develop tools and strategies for the anti-stigma campaign that were acceptable to adolescents and reflected their local context. It required time, rapport building, sustained regular meetings and discussions with the AEAG. Continuous engagement with key stakeholders throughout the formative phase and the active and direct engagement of research participants in designing intervention components can produce the important groundwork for trials that are meaningful and relevant to the community. The formative phase was helpful to test and make necessary changes to the mHealth application and other research tools. It has also helped to identify potential barriers related to the hesitancy in help seeking by adolescents and to adopt positive and pragmatic strategies to implement the trial.

### Implications

The ARTEMIS formative research included key stakeholders and used qualitative methods which helped to assess implementation feasibility and identify course corrections that were needed to improve intervention acceptability and adoption. Earlier research has also found formative research to be useful in planning interventions that are feasible as well as sustainable [49, 50]. While undertaking such formative work is an intensive task and requires time as well as resources, it is expected that the refined intervention informed by the formative learnings and findings will result in improved recruitment of participants, minimise loss to follow-up and improve intervention implementation.

### Electronic supplementary material

Below is the link to the electronic supplementary material.

Supplementary Material 1

## Data Availability

The datasets used and/or analysed during the current study are available from the corresponding author on reasonable request.

## Abbreviations

mhGAP: Mental health gap action programme
EDSS: Electronic Decision Support System
ASHA: Accredited Social Health Activist
AEAG: Adolescent Expert Advisory Group
IEC: Information education communication
ToC: Theory of Change
PWLE: people with lived experience
UPHC: Urban Primary Health Centre
